# Bioactive Tetracalcium Phosphate Scaffolds Fabricated by Selective Laser Sintering for Bone Regeneration Applications

**DOI:** 10.3390/ma13102268

**Published:** 2020-05-14

**Authors:** Tian Qin, Xiaoqian Li, Hui Long, Shizhen Bin, Yong Xu

**Affiliations:** 1 State Key Laboratory of High Performance Complex Manufacturing, College of Mechanical and Electrical Engineering, Central South University, Changsha 410083, China; 163701007@csu.edu.cn (T.Q.); xiaoqianli1952@163.com (X.L.); 2 College of Mechanical and Control Engineering, Guilin University of Technology, Guilin 541004, China; 3 School of Intelligent Engineering, Shaoguan University, Shaoguan 512000, China; longhui32603260@126.com; 4 Research Institute of Light Alloys, Central South University, Changsha 410083, China; 5 Key Laboratory of Hunan Province for Efficient Power System and Intelligent Manufacturing, College of Mechanical and Energy Engineering, Shaoyang University, Shaoyang 422000, China

**Keywords:** bioactivity, tetracalcium phosphate, selective laser sintering, scaffold

## Abstract

Tetracalcium phosphate (TTCP), a potential biological scaffold material, has attracted increasing interest for bone regeneration applications due to its good biodegradability and biocompatibility. In this research, three-dimensional porous TTCP scaffolds were manufactured via selective laser sintering (SLS), and an in-depth and meticulous study on the influence of laser power on the microstructure and mechanical properties of TTCP scaffolds was performed. The results showed that the TTCP particles fused together and formed a solid object due to the decrease in the number of micro-pores in the scaffold as the laser power increased from 6 W to 9 W. The maximum compressive strength that the scaffold could withstand and the strength of the fracture toughness were 11.87 ± 0.64 MPa and 1.12 ± 0.1 MPa·m^1/2^, respectively. When the laser power increased from 9 W to 10 W, the TTCP grains grew abnormally, resulting in diminished mechanical properties. The bioactivity tests showed that the surfaces of the scaffolds were entirely covered by bone-like apatite layers after soaking in simulated body fluid (SBF) for three days, indicating that the scaffolds exhibit excellent bioactivity. Moreover, cell experiments showed that the TTCP scaffolds had good biocompatibility. This study indicated that SLS-fabricated TTCP scaffolds may be a promising candidate for bone regeneration applications.

## 1. Introduction

Bone has a self-healing ability. However, regeneration cannot be completed when bone defects are overlarge [1]. Bone scaffolds offer a promising new approach for bone regeneration, because they can provide a matrix for cell attachment, cell proliferation, and new tissue regeneration [2,3,4]. An ideal scaffold material should satisfy certain conditions, such as good biodegradability, biocompatibility, and bioactivity, and proper mechanical properties [5,6,7].

Tetracalcium phosphate (TTCP), a potential biological scaffold material, has attracted increasing interest for bone regeneration due to its good biocompatibility and biodegradability. Surprisingly, only very little research was done investigating TTCP, and it was about the coating or the bulk material itself. Little attention was paid to the microstructural response trend on the sintering power and assessing the bioactivity of TTCP. TTCP is mainly composed of calcium (Ca) and phosphorus (P), which are the primary components of the bone matrix. In addition, an alkaline environment is formed when TTCP is dissolved in an aqueous solution, which is beneficial for cell adhesion and bone formation [8]. During implantation, the TTCP scaffold surface exhibits a negative charge, calcium ions in the SBF are attracted and form calcium-rich amorphous calcium phosphate (ACP). The positively charged Ca-rich ACP further attracts HPO_4_^2−^ and OH^−^ ions and reacts with them to form a Ca-poor ACP layer. Finally, apatite layers form on the TTCP surface. The surfaces of the scaffolds were entirely covered by bone-like apatite layers after soaking in simulated body fluid (SBF) for three days. Apatite plays an important role in early bone formation and leads to gap/interface healing [9]. These advantages make TTCP a promising scaffold material for bone regeneration.

In addition to having good biological properties, an interconnected porous structure and a high porosity are also important to scaffolds because these characteristics allow cell ingrowth, vascularization, and nutrient delivery [10,11,12,13] Conventional fabrication techniques, such as melt molding, freeze drying, and foam replication, have been used to produce scaffolds. However, these techniques cannot precisely control pore size and porosity. Selective laser sintering (SLS) is a leading technology, which is based on the rapid prototyping method of layer-by-layer manufacturing technology, and can overcome the abovementioned problems encountered in conventional methods [14,15]. This layered manufacturing technique allows the fabrication of porous scaffolds, with precise control of pore size, interconnectivity, porosity, and external geometries [16,17]. Williams et al. fabricated 3D polycaprolactone (PCL) scaffolds with high porosity and interconnected pores via SLS. Their results showed that the porous scaffold had sufficient mechanical properties [18]. Du et al. used SLS to fabricate porous osteochondral scaffolds that consisted of the PCL and hydroxyapatite (HAP). The scaffolds possessed interconnected porous structures that supported cell adhesion and proliferation and induced early bone regeneration [19]. Therefore, SLS is an ideal approach for fabricating interconnected porous bone scaffolds.

Considering the great potential of TTCP in the application of bone scaffolds and the unique advantages of SLS technology in the construction of porous bone scaffolds, herein, the construction of TTCP porous bone scaffold using SLS technology was reported for the first time. The sintering process parameters were optimized. The microstructures of TTCP scaffolds and the microstructural response trend to the sintering power were studied by scanning electron microscopy (SEM). The evaluation of the influence of the sintering power on the performance of the TTCP scaffold mechanics is based on the analysis of the strength of the compressive force, fracture toughness, and hardness. In addition, the scaffolds were placed in SBF, and after a period of immersion, the ability to form apatite on the surface of the SBF was examined and verified by Fourier transform infrared (FTIR) spectroscopy.

## 2. Materials and Methods

### 2.1. Materials and Fabrication

The TTCP powder was purchased from Kunshan China Science and Technology New Materials Co., Ltd. (Kunshan, China) The manufacturing process was as follows: TTCP powder was fabricated by heating a mixture of CaCO_3_ and Ca (H_2_PO_4_) H_2_O to 1500 °C for 18 h. Then, the product was rapidly quenched to indoor temperature in a vacuum drier (SHKTYQ, Shanghai, China). The sintered product was broken into pieces and passed through a 10 μm sieve. The TTCP powder was dried at 90 °C and kept in a vacuum oven (T-LONG, Zhengzhou, China). X-ray diffraction (XRD) verified that the powder was phase-pure TTCP.

### 2.2. Selective Laser Sintering

**Figure 1:**
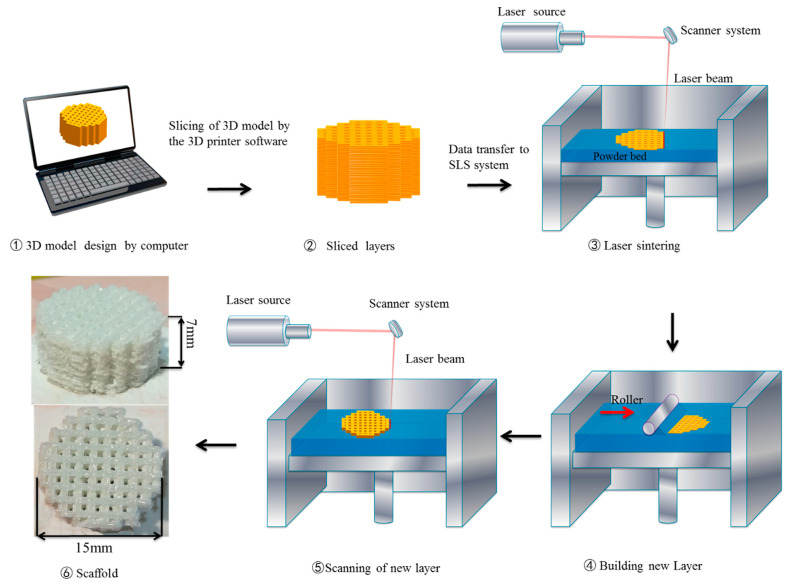
Simplified schematic of the selective laser sintering (SLS) process.

As we know, SLS is a technology that can be used to create three-dimensional porous objects [6,7,20]. The main technique is to use a carbon dioxide laser beam to sinter a powder bed. The SLS machine was mainly constituted by a 100 W Carbon dioxide laser (RAYTO LASER, Jiangsu, China), a three-axis linkage sintering platform, and a control system. In this technology, three-dimensional porous supports are mainly manufactured layer-by-layer from a StereoLithography (STL) file [2,20]. During the SLS manufacturing process, the laser beam scans the powder in surface-based data contained in the slice. The laser beam increases the temperature of the powder to the melting point and makes the particles fuse together to build a solid object. Then, a roller directly places a new powder layer on top of the sintered layer. The above steps are repeated until a 3D scaffold is fabricated successfully. TTCP scaffolds were fabricated by the following parameters: laser power of 6, 7, 8, 9, and 10 W (the scanning speed was 60 mm/min during both acceleration and deceleration periods); laser beam spot diameter of 1 mm; scan line spacing of 2 mm; and layer thickness of 0.1 mm. Figure 1 shows the process of the SLS.

### 2.3. Characterization

The phase compositions of TTCP powder and a scaffold fabricated at a sintering power of 9 W were identified by an X-ray diffractometer (D8-ADVANCE, Bruker AXS Inc., Madison, WI, USA), with a Cu Kα radiation source operating at 40 kV. The obtained peaks were compared with a standard pattern of tetracalcium phosphate (JCPDS NO.25-1137). When the TTCP scaffold was soaked in the SBF, a layer of the mineral material formed on the surface of the scaffold. FTIR was used for detection (Nicolet TM 6700 spectrometer; ThermoScientific Co., Waltham, MA, USA).

### 2.4. Microstructures

TTCP was etched with a 10% hydrofluoric acid solution for 2 minutes. The TTCP scaffolds used here were sintered at 6, 7, 8, 9, and 10 W. The different laser-power-sintered TTCP powders and supports were placed on the sputtering machine (JFC-1600, Jeol Co., Tokyo, Japan) and gold plated for 120 s (JEOL, JFX-1300). SEM (JEOLJSM 7600F, Tokyo, Japan) was used to analyze the microstructures of the TTCP powders and the TTCP scaffolds sintered at different powers. Energy dispersive spectroscopy (EDS) (Hillsboro, OR, USA) was performed for chemical microanalysis.

### 2.5. Mechanical Properties

For the determination of the compressive strength of the TTCP scaffold, a general mechanical tester was used (WD-01, Shanghai Zhuoji Instruments, Shanghai, China). During testing, the maximum load was 100 N, and the displacement rate was 0.5 mm∙min^-1^. Equation (1) can be used to calculate the compression strength:

σ_cmax_ = P_cmax_/A, (1)

where the compressive strength, the maximum failure load, and the cross-sectional area were denoted σ_cmax_, P_cmax_, and A, respectively. The average values of compressive strength were obtained from ten tests for each laser power.

The fracture toughness and the Vickers hardness of TTCP scaffolds were tested with a Vickers microhardness tester (Beijing Optical Instrument Co. Ltd, Beijing, China), and the surfaces of TTCP scaffolds were polished. During testing, the applied load was 2.94 N and the duration was 15 s to induce cracks and indentations. Equation (2) can be used to calculate the fracture toughness [21]:

(2) $$K_{IC}=0.0824(P/C^{3/2}),$$

where the fracture toughness, the induced radial crack length, and the indentation load were denoted *K_IC_*, *C*, and *P*, respectively. The average values of fracture toughness, Vickers hardness, and compressive strength were obtained from ten tests for each laser power.

### 2.6. Bioactivity Test

The TTCP scaffold was soaked in SBF, and its biological activity was observed. The composition and concentration of SBF are similar to those of human plasma. The TTCP scaffolds were immersed in SBF in an incubator at 37 °C for 1, 2, 3, and 4 days. The SBF was replaced every day. After soaking, alcohol was used to clean the scaffolding, which was then placed in air for drying [22,23]. The apatite formed on the surface of TTCP was observed by scanning electron microscopy. In addition, EDS was used to define the elemental constitution of the deposits on the scaffolds after cultivation in SBF. The formation of apatite was analyzed by FTIR.

### 2.7. Cytocompatibility

MG-63 cells were selected to evaluate the cytocompatibility of scaffold materials. In Dulbecco’s Modified Eagle’s Medium (DMEM, Gibco, Germany) supplemented with 10% fetal bovine serum (FBS) and 1% penicillin/streptomycin sulfate, cells were cultured at a density of 1 × 10^5^ cells/scaffold under a humidified atmosphere containing 5% CO_2_. The medium was replaced every two days. All instruments and scaffolds were UV-sterilized for 1 h in advance. After culturing for a predetermined period of time, the cell/scaffold complex was collected from the medium and rinsed three times with phosphate buffer solution (PBS). Subsequently, the cell/scaffold complex was fixed with 3% glutaraldehyde for 30 min and dehydrated with gradient ethanol. Cell viability was analyzed by staining live and dead cells with calcein AM and propidium iodide (PI), respectively. Then, optical analysis was performed through a fluorescence microscope (Olympus Co. Ltd., Tokyo, Japan) equipped with a digital camera (Olympus America Inc., Mel-ville, NY, USA).

## 3. Results and Discussion

### 3.1. Microstructural Evolution

**Figure 2:**
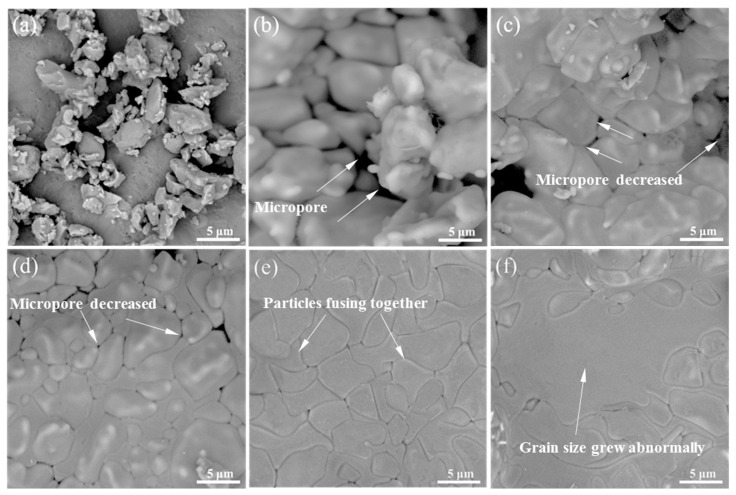
SEM micrographs of the (**a**) tetracalcium phosphate (TTCP) particles and the TTCP scaffolds fabricated at laser powers of (**b**) 6 W, (**c**) 7 W, (**d**) 8 W, (**e**) 9 W, and (**f**) 10 W.

The microstructures of the TTCP powder and the sintered TTCP scaffolds with HF etching are displayed in Figure 2. The TTCP particles were irregular and had rough surfaces (Figure 2a). At a laser power of 6 W (Figure 2b), few TTCP particles had melted and fused together due to the insufficient energy of the laser power. Increasing the power of the laser to 7 W and 8 W (Figure 2c,d) resulted in more particles fusing together, and some micro-pore gaps decreased. These micro-pores resulted from an incomplete fusion of the powders. While the laser power increased to 9 W or greater, the micro-pores diminished, and a compact structure was obtained (Figure 2e,f). The grain size of the scaffold sintered at 10 W grew abnormally compared to that of the scaffold sintered at 9 W. According to related research [24,25,26], abnormally grown grains may adversely affect the compactness of the scaffold, and to some extent affect the mechanical properties of the scaffold. For example, Tolouei’s study found that abnormally grown grains caused micro-pores in the microstructure, and further led to the deterioration of mechanical properties, which also confirmed this view [27]. Therefore, from the standpoint of the microstructure of the scaffold, the optimal power for preparing the scaffold is 9 W. 

### 3.2. Mechanical Properties

**Figure 3:**
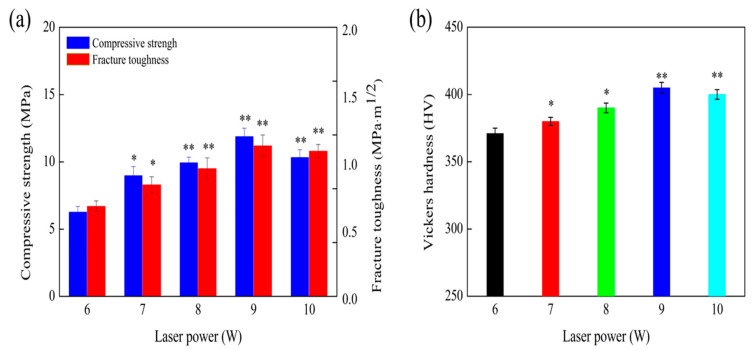
(**a**) Compressive strength and fracture toughness and (**b**) Vickers hardness of TTCP scaffolds sintered at 6, 7, 8, 9, and 10 W.

Figure 3 shows the compressive strength, fracture toughness, and Vickers hardness of the TTCP scaffolds sintered at 6, 7, 8, 9, and 10 W. The change in the laser power was from 6 W to 9 W. The optimum values of compressive strength and fracture toughness for these samples were 11.87 ± 0.64 MPa and 1.12 ± 0.1 MPa·m^1/2^, respectively, which was due to the reduction in micro-pores and the increased compactness of the structure. However, the compressive strength and fracture toughness of TTCP were considerably lower than the compressive strength (between 90 and 224 MPa) and fracture toughness (2–12 MPa·m^1/2^) of cortical bone [28,29]. In the research, as the power of the laser increased, the compressive strength and fracture toughness of the scaffold decreased accordingly because of the abnormal grains that developed during the growth process [30]. The Vickers hardness was 371 HV at 6 W and 405 HV at 9 W. However, the Vickers hardness decreased to 400 HV at 10 W. The hardness can reflect the elastic and plastic deformation resistance of TTCP [31]. Therefore, TTCP scaffolds sintered at 9 W had the best mechanical properties among the tested scaffolds.

**Figure 4:**
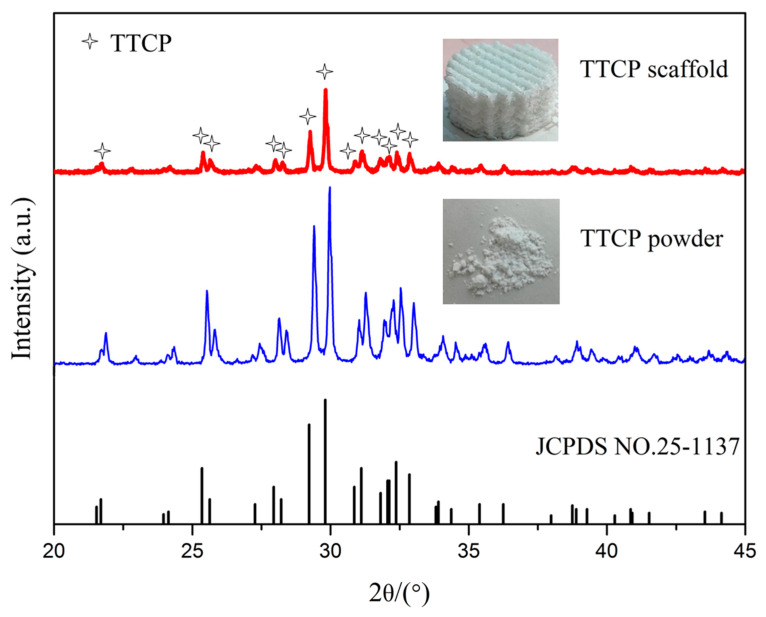
XRD diffraction patterns of the TTCP powders and the scaffold fabricated at 9 W.

A porous TTCP scaffold with optimal mechanical properties was fabricated by SLS at a laser power of 9 W. The top view and isometric view of the scaffold are displayed in Figure 1. The diameter of the scaffold was 15 mm, and the height was 7 mm. Figure 4 shows the XRD pattern of the TTCP powder and the TTCP scaffold fabricated at a laser power of 9 W. The results showed that the characteristic peaks of the powder and bracket at 21.8°, 25.4°, 25.7°, 28.0°, 28.3°, 29.3°, 29.8°, 30.9°, 31.2°, 31.9°, 32.1°, 32.4°, and 32.9° corresponded to (121), (200), (130), (211), (211), (032), (040), (−103), (221), (−132), (113), (212), and (−212) crystal plane reflection, respectively, which is consistent with the standard card of TTCP (JCPDS NO.25-1137). Only TTCP peaks were detected in the XRD patterns, indicating that no phase evolution or transformation occurred in the SLS process.

### 3.3. Bioactivity Tests

**Figure 5:**
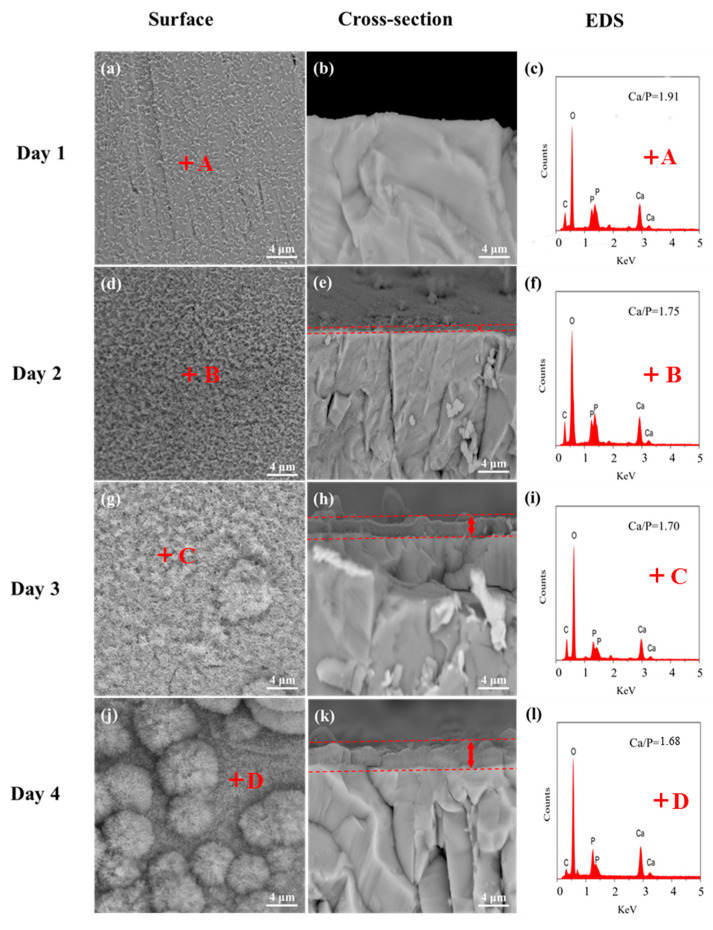
SEM images (surface and cross-section) and EDS traces of the TTCP porous scaffolds after soaking in SBF for different durations: (**a**–**c**) one day, (**d**–**f**) two days, (**g**–**i**) three days, and (**j**–**l**) four days.

The TTCP scaffolds sintered at 9 W were chosen as specimens for the bioactivity test. The surface and transverse section views and EDS spectra of the TTCP scaffolds after soaking in the SBF for 1, 2, 3, and 4 days are displayed in Figure 5. After one day of immersion, some worm-like crystals precipitated and uniformly distributed on the surfaces of the scaffolds; these crystals were few in number, and the matrix surface can be seen clearly (Figure 5a). After two days, the number of worm-like crystals increased, and the matrix surface was still visible (Figure 5d). After three days, the surface of the scaffolds was completely covered with an apatite layer, and the deposited layer became denser (Figure 5g). After four days, the layer became thicker and denser, and the apatite crystals agglomerated, similar to cauliflower clusters (Figure 5j). This cross-sectional view shows the TTCP scaffold soaked in SBF for one, two, three, and four days in Figure 5b,e,h,k, respectively. When the TTCP scaffolds were soaked in SBF, an apatite layer with a continuous thickness formed on its surface. The apatite layer became thicker as the soaking time increased. The EDS spectra of the crystal on the TTCP surface mainly contained P, Ca, C, and O (Figure 5c,f,l). The presence of C indicated the formation of hydroxy-carbonate-apatite. The Ca/P ratio of the TTCP scaffolds was 2 [32]. After one day of soaking, the Ca/P molar ratio of the TTCP scaffolds decreased to 1.91 due to the formation of apatite. The Ca/P molar ratios of the TTCP scaffolds after two, three, and four days of soaking were 1.75, 1.70, and 1.68, respectively. These values were close to the theoretical Ca/P ratio of stoichiometric HA (1.67) [33]. In summary, TTCP exhibited excellent biological activity.

**Figure 6:**
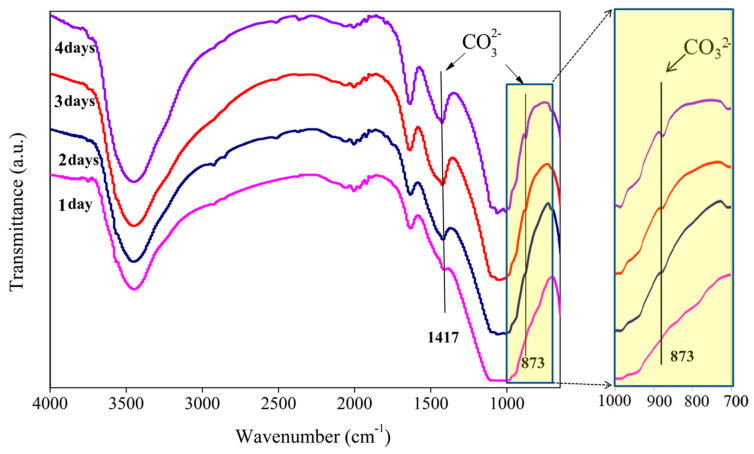
FTIR traces of TTCP soaked in SBF for one, two, three, and four days.

The FTIR (Figure 6) results showed the appearance of a pronounced band at 1417 cm^−1^ and 873 cm^−1^, which is specific to carbonated apatite [9,34]. As the soaking time increased, the carbonate began to appear and then gradually increased. This phenomenon likely occurred because the phosphate was replaced by carbonate during the process of apatite formation.

**Figure 7:**
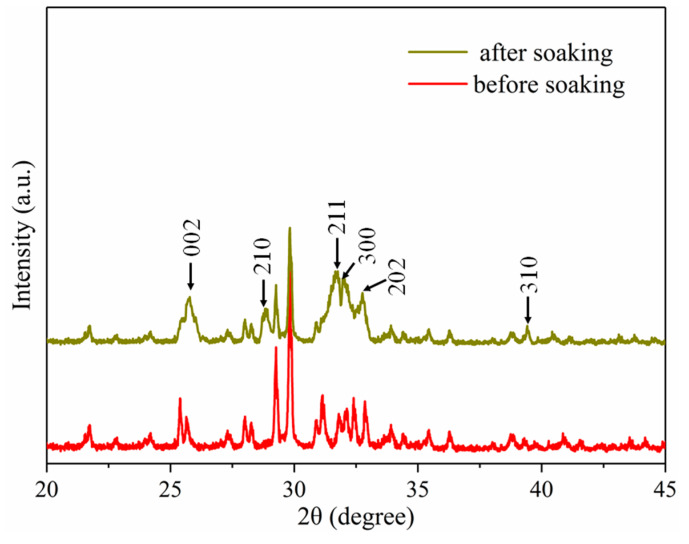
XRD pattern of TTCP scaffold before and after soaking in SBF for four days.

XRD analysis was performed on the TTCP scaffold before and after being immersed in SBF, to evaluate the change in phase composition, thereby confirming the formation of hydroxyapatite; the results are shown in Figure 7. In the XRD patterns, the characteristic diffraction peaks appearing at 25.8°, 28.5°, 31.7°, 32.1°, 32.8°, and 39.5° were attributed to the reflection of the (002), (210), (211), (300), (202), and (310) crystal planes, respectively [35]. These results confirmed the formation of hydroxyapatite on the surface of the TTCP scaffold.

### 3.4. Mechanism of Apatite Formation

**Figure 8:**
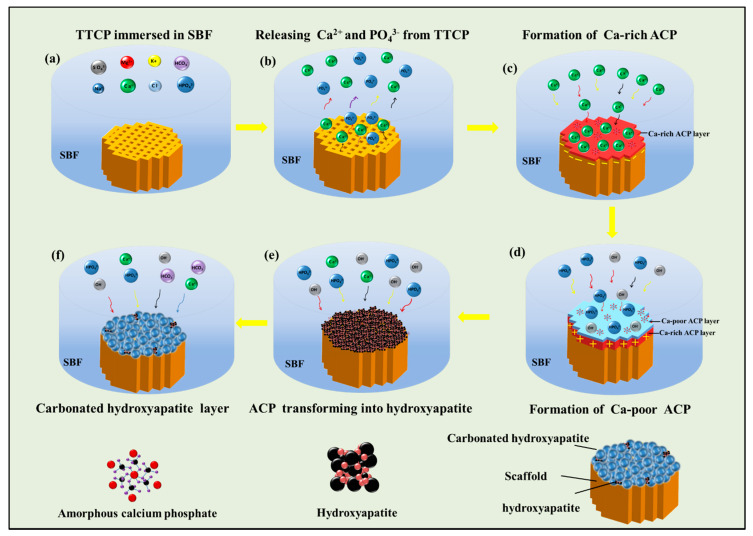
Study on the mechanism of the formation of apatite: (**a**) TTCP immersed in SBF (**b**) dissolution of TTCP, (**c**) formation of Ca-rich ACP, (**d**) formation of Ca-poor ACP, (**e**) increasing formation of apatite on the surface of the TTCP scaffolds, and (**f**) hydroxyapatite containing carbonate in its structure.

Soaking ceramics (with a proper surface) in an SBF solution can induce apatite formation [36]. Figure 8 shows the mechanism of apatite formation on the surface of TTCP when it is immersed in SBF.

① TTCP is the most soluble compound among all the calcium phosphates [34]. After soaking in SBF, the surface of the TTCP scaffold dissolved, and ${\mathrm{Ca}}^{2+}$ and ${\mathrm{PO}}_{4}^{3-}$ ions were released [9,37] (Figure 8b).
  (3) $${\mathrm{Ca}}_{4}{\left({\mathrm{PO}}_{4}\right)}_{2}O+H_{2}O=4{\mathrm{Ca}}^{2+}+2{\mathrm{PO}}_{4}^{3-}+2{\mathrm{OH}}^{-}$$
② The isoelectric point of calcium phosphate ceramics is lower than the PH of SBF, so the surface of TTCP exhibits a negative charge characteristic in its exposed crystal structure [38]. The positively charged calcium ions in the SBF were attracted by the ${\mathrm{PO}}_{4}^{3-}$ ions, and the final ions formed calcium-rich amorphous calcium phosphate (ACP). With the accumulation of Ca^2+^ ions, the TTCP surface gained a positive charge (Figure 8c) [39,40].
  (4) $$10{\mathrm{Ca}}^{2+}+6{\mathrm{HPO}}_{4}^{2-}+8{\mathrm{OH}}^{-}\to {\mathrm{Ca}}_{10}{\left({\mathrm{PO}}_{4}\right)}_{6}{\left(\mathrm{OH}\right)}_{2}+6H_{2}O$$
③ The positively charged Ca-rich ACP further attracts HPO_4_^2−^ and OH^−^ ions and reacts with them to form a Ca-poor ACP layer (Figure 8d) [39,40].
④ Eventually, the ACP layer transforms into a crystalline apatite layer, which is relatively stable [40]. Ca^2+^, HPO_4_^2−^, and OH^−^ ions are then absorbed by the apatite on the TTCP surface by electrostatic attraction and chemical bonding, and an increasing amount of apatite forms on the surfaces of the TTCP scaffolds (Figure 8e) [39,40].
⑤ After formation, the apatite grew and became spherical by consuming Ca^2+^, HPO_4_^2−^, OH^−^, and HCO_3_^−^ from the SBF [38,40] (Figure 8f). Each spherulite was composed of a large number of flakes that aggregated into a petal shape. The flake was hydroxyapatite and contained carbonate in its structure [38,40].

### 3.5. Cytocompatibility

**Figure 9:**
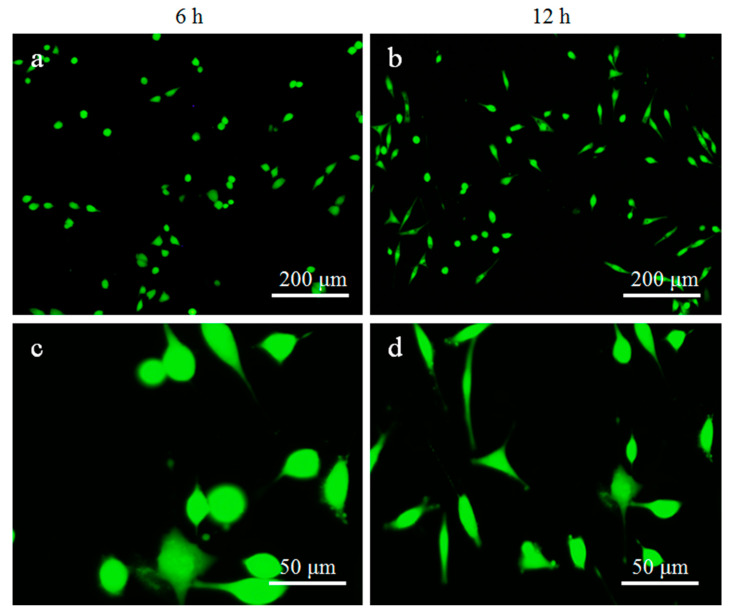
Viability analysis of MG63 cells after cultivation on TTCP scaffold for 6 and 12 h. (**a**,**b**) Low magnifications images and (**c**,**d**) high magnification images.

The viability of MG-63 cells was evaluated by immunofluorescence, and the results were shown in Figure 9. After 6 h of culture, cells began to stretch and appear flat, as shown in Figure 9a. As the culture time was extended to 12 h, most of the cells showed a fusiform morphology and had a small number of filamentous pseudopodia, as could be seen in Figure 9b,d. These results indicated that the bone scaffold material used had good biocompatibility, which is conducive to cell adhesion and proliferation on the scaffold.

## 4. Conclusions

Porous TTCP ceramic scaffolds were fabricated by SLS at 6, 7, 8, 9, and 10 W in this paper. When the power of the laser increased from an initial 6 W to 9 W, the TTCP particles gradually fused together. As a result, the compressive strength and fracture toughness of the scaffold could be effectively improved. The optimum fracture toughness and compressive strength were 1.12 ± 0.1 MPa·m^1/2^ and 11.87 ± 0.64 MPa, respectively, and the peak hardness was 405 GPa. After one day of soaking in SBF, nano-apatite was produced on the surfaces of the TTCP scaffolds. After three days, the surface of the scaffolds was completely covered with an apatite layer, which showed good bioactivity. Furthermore, the biocompatibility of TTCP scaffolds was evaluated by cell culture, verifying the TTCP scaffolds had good biocompatibility. The TTCP scaffold, fabricated via SLS, is a promising candidate for bone tissue applications.
